# A robust algorithm for computational floating body dynamics

**DOI:** 10.1098/rsos.231453

**Published:** 2024-04-03

**Authors:** J. Roenby, S. Aliyar, H. Bredmose

**Affiliations:** ^1^ Stromning Aps, Luftmarinegade 62, København K 1432, Denmark; ^2^ Department of Science and Environment, Roskilde University, Universitetsvej 1, Roskilde 4000, Denmark; ^3^ Department of Wind and Energy Systems, Technical University of Denmark, Nils Koppels Alle, Kgs. Lyngby 2800, Denmark

**Keywords:** numerical methods, added mass, computational fluid dynamics, OpenFOAM, FloatStepper, floating body dynamics

## Abstract

We present a non-iterative algorithm, FloatStepper, for coupling the motion of a rigid body and an incompressible fluid in computational fluid dynamics (CFD) simulations. The purpose of the algorithm is to remove the so-called added mass instability problem, which may arise when a light, floating body interacts with a heavy fluid. The idea underlying the presented coupling method is to precede every computational time step by a series of prescribed probe body motions in which the fluid response is determined, thus revealing the decomposition of the net force and torque into two components: (i) an added mass contribution proportional to the instantaneous body acceleration and (ii) all other forces and torques. The algorithm is implemented and released as an open-source extension module to the widely used CFD toolbox, OpenFOAM, as an alternative to the existing body motion solvers. The accuracy of the algorithm is investigated with several single-phase and two-phase flow benchmark cases. The benchmarks demonstrate excellent stability properties, allowing simulations even with massless bodies. They also highlight aspects of the implementation, such as the mesh motion method, where it can be improved to further enhance the flexibility and predictive capabilities of the code.

## 1. Introduction

Accurate modelling of floating body motion is important for the design of offshore structures. This requires a robust numerical approach to both free surface flow and wave-structure interaction. While many basic response effects are well-described by linear and second-order radiation-diffraction theory [1], this relies on the assumption of small wave steepness and small body motion. To describe response effects from large waves, slamming from breaking waves, green water flow on the structure and viscous damping, more accurate modelling is needed.

During the last decades, Computational Fluid Dynamics (CFD) for free surface flow has matured to a level where it is now a viable solution for engineering calculation of design wave events. Among the various CFD methodologies, the Finite Volume Method (FVM) in combination with the Volume of Fluid technique (VoF) for the free surface treatment [2] has shown robust performance with the ability to calculate for example breaking wave loads on monopiles [3–5]. Several publications have shown good comparisons to experimentally measured force and pressure for such breaking wave impacts; for example, [6]. Compared with potential flow solvers, CFD includes the calculation of viscous effects accounting for drag and vortex shedding combined with complex free surface behaviour. Much research effort has gone into the detailed VoF schemes to avoid numerical smearing of the air–water interface. Well-known methods after the original paper of Hirt and Nichols [2] include the works in [7,8], as well as the isoAdvector scheme [9], which can be applied on unstructured meshes.

Given the successful results for fixed structures, the application of finite volume CFD to floating body motion appears to be a natural next development. In principle, the body motion can be treated by calculating its acceleration in each time step through Newton’s second law, with the surface integrated fluid pressure on the force side of the equation. Several studies with FVM-VoF-based floating body CFD have been published in recent years, especially within ship motion, wave energy generation and floating wind turbine motion; for example, [10–14]. A thorough review of various solver types is given by Windt *et al*. [15]. Furthermore, Ransley *et al*. [16] presented a comparative study for the response of focused wave groups for a hemispherical-bottomed buoy and a truncated cylinder with a cylindrical moon-pool with both potential flow solvers and CFD. A straightforward implementation of the above steps, however, has shown to be susceptible to the added mass instability for bodies with low structural mass. When the body is rigid, and the fluid is incompressible, an acceleration of the body must be accompanied by a simultaneous acceleration in the surrounding fluid. As such, their coupling is infinitely tight due to the kinematics of the problem. Any time lag in the numerical treatment of this simultaneous body-fluid motion opens the door for unphysical injection of momentum into the system. This is exactly what happens in the added mass instability, which can degrade simulation accuracy and lead to sudden simulation crash. We stress that the instability is caused by the incompressibility condition and as such, any solver, CFD or potential, treating body and fluid motion sequentially may suffer from it. One would not expect the instability to be dominant in simulations where compressibility (real or artificial) is introduced in the solution of the fluid equations. Indeed, from private communication with researchers using the Smoothed-Particle Hydrodynamics (SPH) method, the added mass instability does not seem to be a problem for this type of artificial compressibility-based solver.

The need for a proper treatment of added mass in floating body CFD has been discussed already by Söding [17] in a conference paper, which appears to have only little recognition. Bettle [18] discussed the stability problem in the context of CFD for submarine manoeuvring and devised a coupling algorithm with iterations between body and fluid motion. A floating body solver along the lines of Bettle’s work is found in the widely used open-source CFD code, OpenFOAM. This was improved in the work of Dunbar *et al*. [19] and Chow *et al*. [20] using dynamic relaxation techniques, and by Bruinsma *et al*. [21] who stabilized solutions by relaxing fluid pressure in the iteration loop. The latter concluded that more work is needed to achieve a robust solution for the added mass problem since the stabilization techniques lead to larger computational effort. Further steps in the solution of the added mass problem have been proposed by Devolder *et al*. [22] in terms of an acceleration technique for the added mass iterations with 1 degree of freedom (DoF) and by Veldman *et al*. [23] in terms of an approximate initial added mass term.

While the iterative methods can give accurate results upon convergence, a robust solution of the equations of motion requires separation of the added mass force from the overall fluid force and lumping the body and added mass together to properly isolate the acceleration in the force equation. This is the core idea of the present paper. We develop an algorithm, where the added mass is determined explicitly in each time step and thus allows for an accurate and direct calculation of the true body acceleration without the need for outer iterations. We implement the algorithm and demonstrate its robustness through a series of numerical experiments.

It is worth noting that the idea of an explicit added mass matrix in floating body modelling has also been presented by other researchers. In this respect, our approach has strong similarities to the algorithm outlined by Söding [17] and applied by Shigonov *et al*. [24] for aircraft landing on water in 3 DoF’s. In Söding’s paper, the need for an explicit added mass matrix for numerical stability is explained, and an iteration-based method for its determination is formulated. In a study by Meyer *et al*. [25], this algorithm was applied to calculate the motion of a yacht in head waves. To the best of our knowledge, no thorough demonstration of the stability properties exists in the literature, and no open-source CFD implementation is available to the scientific community.

The contribution of the present work is to develop and implement in a fully parallelized unstructured FVM CFD code, OpenFOAM, a 6-DoF added mass aware algorithm, which in contrast to earlier works, is free of outer iteration loops. We analyse and demonstrate its stability properties and validate it against five test cases. Two of these have analytical solutions, a rising disc and a wiggling ellipse, and two contain comparisons to experimental results, namely, a freely floating box and a moored box in regular waves. We hope that the new robust method, and the release of the implementation as an OpenFOAM extension module [26], will provide a simpler approach to CFD simulations of floating body problems.

## 2. The added mass instability problem

We consider the numerical coupling of a floating rigid body with a surrounding incompressible fluid. The fluid may either be a single fluid or two immiscible fluids separated by a sharp fluid interface. It may either be inviscid or viscous with slip or no-slip boundary condition on the body so the instantaneous distribution of fluid pressure—and possibly shear stress—exerts a force and a torque on the body.

When an external force, F , such as gravity or a spring works on a rigid body immersed in fluid, the body will accelerate and so must the surrounding fluid to accommodate the body displacement. In a small time step, δ⁢t , the momentum of the body-fluid system will increase by Fδt , and from a numerical point of view, the difficulty is that we do not know *a priori* how this increase in system momentum is distributed between the fluid and the body. It is the task of a coupling algorithm to find this distribution and advance the system accordingly.

**Figure 1.**
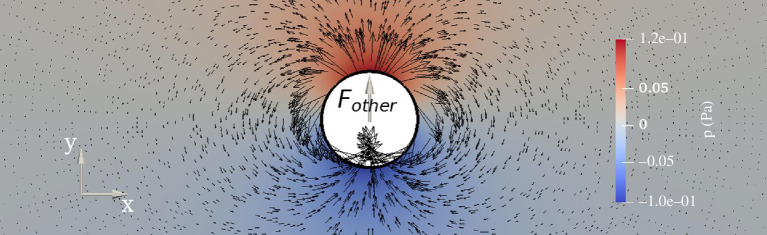
Pressure and velocity field around circular body exposed to a force along the y -axis.


For illustrative purposes, let us consider a rigid cylinder floating in an unbounded two-dimensional ideal fluid of uniform mass density $\rho_{f}$ , as shown in figure 1. The body has radius R and uniform mass density $\rho_{b}$ , and hence, mass (per unit length) $m_{b}=\rho_{b}\,\pi \,R^{2}$ . If the body is exposed to a net force, F , along the y axis, this can be written as

(2.1) $$F=F_{\text{other}}-m_{a}\,a,$$

where *m*_*a*_ is the added mass of the body, a is the instantaneous y -acceleration of the body and $F_{\text{other}}$ represents all other forces on the body (gravity, buoyancy, mooring lines etc.). In this simple example, the added mass is known analytically to be $m_{a}=\rho_{f}\,\pi \,R^{2}$ . Equating the total force, F , to $m_{b}\,a$ and isolating *a*, we get the body acceleration,


(2.2) $$a=\frac{F_{\text{other}}}{m_{a}+m_{b}}.$$

In CFD simulations, we often do not know *m*_*a*_ and/or $F_{\text{other}}$ . Therefore, in partitioned coupling algorithms, we typically resort to iteration between

1. calculating the body acceleration as $a=F/m_{b}$ (or some relaxed variant of this), where F includes the force from the surrounding fluid flow, and
2. calculating the fluid flow and force on the body, F , resulting from the body acceleration, a .

The hope is that iterations between these two steps will eventually converge to the physically correct body acceleration and fluid response, which is assumed to be reached when reiteration no longer changes the results (to within a tolerance). It is, however, well-known that this iterative procedure is unstable when the body mass is smaller than the added mass [27].

Let us first consider a loose body-fluid coupling algorithm without any iterations. Each time step contains a single update of the body state followed by an update of the fluid state. We assume that the only force on our circular body is gravity, $g=-g\hat{y}$ , and so, $F_{\text{other}}$ is constant in time. The body state is then represented by the body position and velocity $\left(x_{b},v_{b}\right)$ , here restricted to motion along the y -axis. The fluid state is represented by the velocity field and pressure field (u,p) . The algorithm could look as sketched in Algorithm 1. In the fluid initialization in Step 1 and in Step 7, it is vital to ensure that proper boundary conditions are specified for the velocity and pressure fields on the body boundary since these contain the coupling between body and fluid (will be detailed in §§3.1.1 and 4.6).


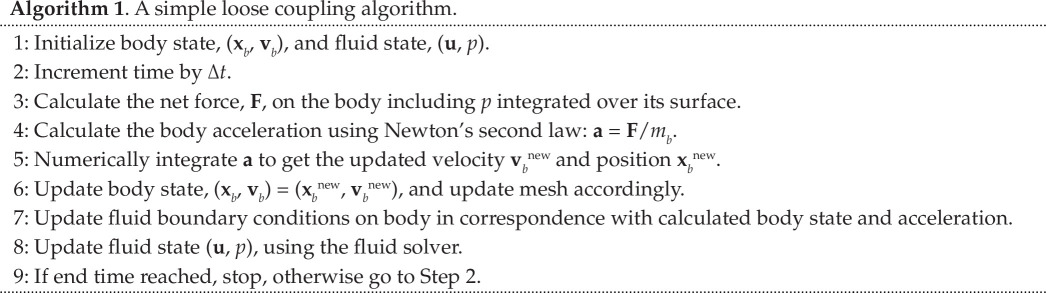


**Figure 2.**
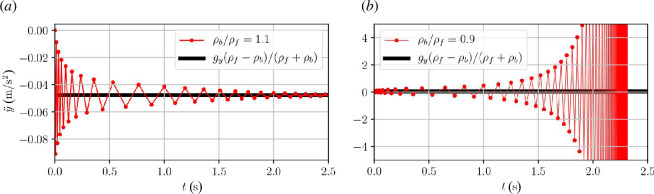
Acceleration of freely floating disc initially at rest in two-dimensional ideal fluid calculated with Algorithm 1. Gravity $g_{y}=-1$ m/s^2^, disc radius R=1 m and circular outer domain radius at 40 m. (*a*) Convergence with $\rho_{b}/\rho_{f}=1.1$ kg/m^3^. (*b*) Divergence with $\rho_{b}/\rho_{f}=0.9$ kg/m^3^. Theoretical acceleration for infinite domain shown in black.

In Step 4, we know—but for now ignore—that part of the force experienced by the body is due to its instantaneous acceleration, cf. equation (2.1). Even if we did not know the specific values of $F_{\text{other}}$ and *m*_*a*_, we can still explore the implications of ignoring the added mass force in Step 4. Using equation (2.1), we have


(2.3) $$a_{n+1}=\frac{F_{\text{other}}}{m_{b}}-\frac{m_{a}}{m_{b}}a_{n},$$

where the subscript n indicates time step. If we call $F_{\text{other}}/m_{b}=a_{0}$ , insert the corresponding expression for *a*_*n*_ in terms of $a_{n-1}$ , and so forth until we reach n=0 , we get


(2.4) $$a_{n+1}=a_{0}\sum_{k=0}^{n}{\left(-\frac{m_{a}}{m_{b}}\right)}^{k}\to \left\{ \begin{array}{ll} \frac{F_{\text{other}}}{m_{b}+m_{a}} & \text{if }m_{a}<m_{b},\\ \pm \infty & \text{if }m_{a}>m_{b}, \end{array}\right.$$

that is, an alternating geometric series bound to diverge in an alternating manner when the added mass exceeds the body mass. This is the added mass instability in a nutshell. It is an inherent problem in any partitioned coupling mechanism [28], and many codes exhibit the instability. This also includes the most widely used open-source CFD code, OpenFOAM [19,22,29], which we use in this work as our implementation platform. Figure 2 illustrates the change from stable to unstable solver behaviour when the body becomes lighter than the surrounding fluid in the case of a circular body accelerating in a fluid due to gravity. The simulations were run with OpenFOAM’s interFoam solver for the fluid motion [8] and the sixDoFRigidBodyMotion module [30] for body motion. Both the converging and diverging solution in figure 2 exhibit an alternating overshoot, where the alternation is caused by the factor ${\left(-1\right)}^{k}$ in equation (2.4). Physically this can be understood by noting that an overestimated body acceleration in one direction will cause an overestimated pressure response force on the body, leading to overestimated body acceleration in the opposite direction and so forth.

To circumvent the added mass instability, many codes, including OpenFOAM, introduce an outer corrector loop for stronger coupling between body and fluid solution within each time step. In these new iterations, an under-relaxed acceleration is used as shown in Algorithm 2.


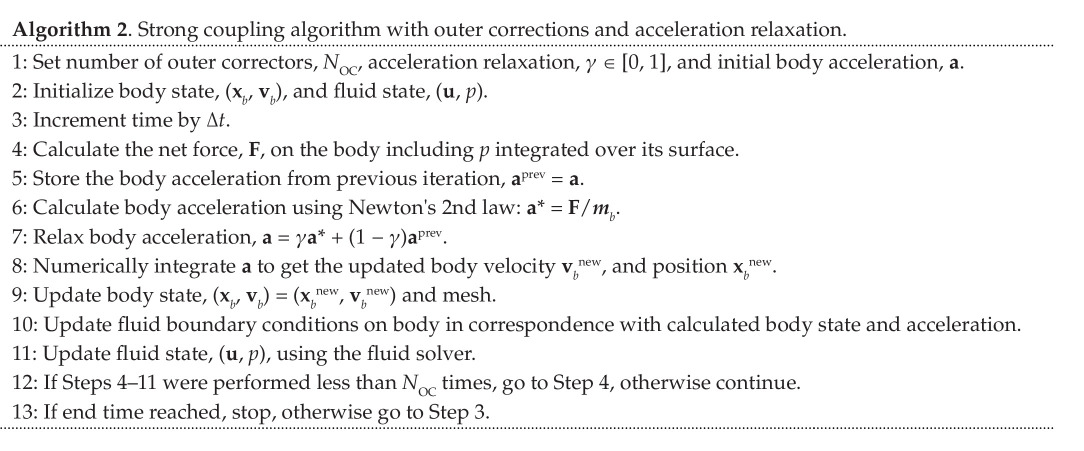


The introduction of the acceleration relaxation factor, γ∈[0,1] , modifies the iterative process in equation (2.3) to


(2.5) $$a_{n+1}=\gamma \left(a_{0}-\frac{m_{a}}{m_{b}}a_{n}\right)+(1-\gamma )a_{n},$$

where the subscript is now an iteration counter rather than a time step counter. Tracking this iterative equation back to the zeroth iteration, we get the modified geometric series,


(2.6) $$a_{n}=\gamma a_{0}\sum_{k=0}^{n}{\left[1-\gamma \left(1+\frac{m_{a}}{m_{b}}\right)\right]}^{k}.$$

**Figure 3.**
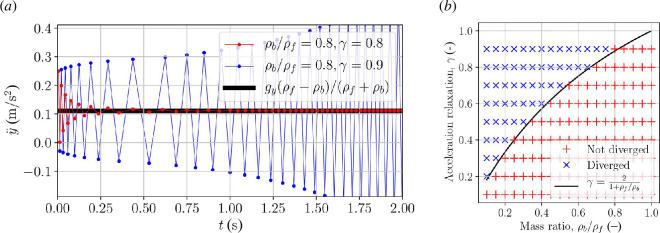
(*a*) As Figure 2, but with $\rho_{b}/\rho_{f}=0.8$ and acceleration relaxation γ=0.8 and 0.9 ( $\gamma_{c}\approx 0.889$ ). (*b*) 171 simulations with $\rho_{b}/\rho_{f}=\left[0.1:0.05:1\right]$ and γ=[0.1:0.1:0.9] . Blue cross indicates simulation crashing due to divergence. Red plus means simulation reached t=3⁢s without crash. Black curve is the theoretical $\gamma_{c}$ from equation (2.7).

This converges if the square bracket has absolute value smaller than 1, leading to the stability criterion,


(2.7) $$\gamma <\gamma_{c}=\frac{2}{1+m_{a}/m_{b}},$$

depending on the instantaneous ratio between added and body mass. This stability criterion was also stated in [17] and derived in a slightly different manner in [22]. It is clear that the under-relaxation procedure enables convergence for body mass smaller than the added mass. Figure 3*a* demonstrates the change to convergence for $\gamma <\gamma_{c}$ in an OpenFOAM simulation of a light circular body rising in a heavy inviscid fluid. Figure 3*b* shows the stability criterion in equation (2.7) plotted together with numerically obtained convergence tests using OpenFOAM for a scan of acceleration relaxation values and density ratios. This clearly demonstrates that the theoretically obtained convergence criterion indeed applies to the specific body-fluid coupling implemented in OpenFOAM.

When the body-to-fluid density ratio approaches 0, so does $\gamma_{c}$ , and hence, also the change in acceleration between iterations diminishes, cf. equation (2.5). In other words, a larger number of iterations is necessary for light bodies. This becomes computationally expensive since each outer iteration involves a full CFD update of the fluid state. Also, for problems involving a body near a free surface, or body motion close to other boundaries, the added mass will vary in time and so, therefore, will $\gamma_{c}$ . There exist methods for dynamic relaxation that have been used with some success [19,20]. Here, we take a different approach: instead of trying to fix the trial-and-error approach to finding a consistent body acceleration, we attempt to exploit the freedom CFD grants us to directly measure the zero-acceleration force, $F_{\text{other}}$ , in a virtual time step before the actual time step is taken. Likewise, we use a simplified version of the CFD solver to obtain the instantaneous added mass. This allows us to calculate the body acceleration directly, which is then used to find the new body velocity and position. In this way, we obtain a coupling mechanism that is completely freed from the added mass instability and outer iterations.

## 3. Governing equations

### 3.1. Fluid motion

The fluid motion is assumed to be governed by the incompressible Navier–Stokes equations. It may be a single phase with constant density, $\rho_{f}$ , or two immiscible phases separated by a sharp fluid interface across which the fluid mass density jumps from the value $\rho^{+}$ in the reference fluid to $\rho^{-}$ in the other fluid. The fluid equations of motion are then


(3.1) $$\frac{\partial \rho }{\partial t}+\nabla \cdot (\rho u)=0,$$

(3.2) ∇⋅u=0,

(3.3) $$\frac{\partial \rho u}{\partial t}+\nabla \cdot (\rho uu)=-\nabla p+\rho g+\nabla \cdot (\mu \nabla u)+f,$$

where g is the gravity vector and f represents other forces such as surface tension and external forces. The fluid state at any time is represented by the density field, ρ , the pressure field, p , and the three components of the velocity field, u . As ρ , the dynamic viscosity, μ , takes constant values, $\mu^{+}$ and $\mu^{-}$ , in the two fluids. Both ρ and μ may be expressed in terms of an indicator function, H(x,t) , which is a Heaviside function taking the value 1 in the reference fluid and 0 in the other


(3.4) $$\rho =\rho^{+}H+\rho^{-}(1-H)\;\text{and}\;\mu =\mu^{+}H+\mu^{-}(1-H),$$

Equation (3.1) can then be replaced by the equivalent equation


(3.5) $$\frac{\partial H}{\partial t}+\nabla \cdot (Hu)=0.$$

This is the starting point for derivations of VoF schemes used to track the sharp fluid interface. For incompressible single-phase flows, ρ is constant in the whole domain, and equation (3.5) is trivially satisfied.

Above, we have used (u,p) to specify the state of the fluid. This is sufficient for single-phase flows. For two-phase flows with a sharp fluid interface, we need to augment u and p with a description of the instantaneous fluid interface position. Since this is encoded in the density field, jumping from one value to another at the interface, we will henceforth use the triplet (ρ,u,p) to represent the fluid state.

#### 3.1.1. Boundary conditions

The gradient of the indicator field, H , is a three-dimensional Dirac δ -function that is 0 everywhere except on the fluid interface, where it is infinite and points along the interface normal, ${\hat{n}}_{I}$ , into the reference fluid,


(3.6) $$\nabla H={\hat{n}}_{I}\delta (x-x_{I}).$$

On domain boundaries, one can therefore specify a desired contact angle or, rather, interface orientation by specifying ∇⁡H . Often this angle is not of practical interest, and one simply uses a zero gradient Neumann boundary condition, ${\hat{n}}_{b}\cdot \nabla H=0$ , on walls and outlets, where ${\hat{n}}_{b}$ is the unit normal of the boundary. For inlet boundaries, one must use a Dirichlet boundary condition to specify the interface position of the inflowing fluid.

For the velocity field, u , we either use a slip condition or a no-slip condition on walls. For slip, we must have ${\hat{n}}_{b}\cdot u={\hat{n}}_{b}\cdot v_{b}$ , where $v_{b}$ is the velocity of the boundary point. The tangential velocity component can be written $u_{t}=(I_{3}-{\hat{n}}_{b}{\hat{n}}_{b})u$ , where *I*_3_ is the 3 × 3 identity matrix and ${\hat{n}}_{b}{\hat{n}}_{b}$ is the outer product of the vector ${\hat{n}}_{b}$ with itself. For slip, a Neumann condition can be applied for the tangential component, ${\hat{n}}_{b}\cdot \nabla u_{t}=0$ . In case of no-slip, the full velocity on the boundary must follow the velocity of the boundary point, $u=v_{b}$ . For inlet boundaries, we must specify the velocity value, and for domain outlets, we can use ${\hat{n}}_{b}\cdot \nabla u=0$ .

For the pressure, boundary conditions are formulated by requiring consistency with the Navier–Stokes equations (3.3) also on the boundaries of fixed and moving walls. Isolating the pressure gradient and dotting with the boundary normal, we get the Neumann boundary condition,


(3.7) $${\hat{n}}_{b}\cdot \nabla p=-{\hat{n}}_{b}\cdot \left(\frac{\partial \rho u}{\partial t}+\nabla \cdot (\rho uu)-\rho g-\nabla \cdot (\mu \nabla u)-f\right).$$

As we will see below, if the boundary is accelerating, care must be taken to encode the acceleration correctly in the implementation of the boundary condition.

### 3.2. Rigid body motion

For the body equations of motion, we start by defining a body-fixed coordinate system centred at some chosen point, $x_{0}(t)$ , and with coordinate axes spanned by the three mutually orthogonal unit vectors $q_{1}(t),q_{2}(t)$ and $q_{3}(t)$ . Together, $x_{0}$ and the orthogonal orientation matrix, $Q=[q_{1}\;q_{2}\;q_{3}]$ , define the instantaneous configuration of the body. For convenience, we will sometimes denote the body configuration with the shorthand notation


(3.8) $$x_{b}=\left[x_{0}\;Q\right].$$

The body translational velocity is


(3.9) $${\dot{x}}_{0}=v_{0}(t),$$

and the rotational velocity is given in laboratory coordinates by $\omega (t)=[\omega_{1}\;\omega_{2}\;\omega_{3}{]}^{T}$ , such that


(3.10) $${\dot{q}}_{i}=\omega \times q_{i}\;\text{for}\;i=1,2,3.$$

If we define for any vector $v=[v_{1}\;v_{2}\;v_{3}{]}^{T}$ , the skew-symmetric matrix


(3.11) $$v\times =\left[\begin{array}{ccc} 0 & -v_{3} & v_{2}\\ v_{3} & 0 & -v_{1}\\ -v_{2} & v_{1} & 0 \end{array}\right],$$

then equation (3.10) can also be written in matrix form as


(3.12) $$\dot{Q}=\omega \times Q.$$

The acceleration equations are given by Newton’s second law


(3.13) $$\frac{dp}{dt}=F,\;\text{and}\;\frac{dL}{dt}=\tau ,$$

where the body linear and angular momentum, force and torque (with respect to the laboratory frame origin) are, respectively,


(3.14) $$p=\int_{\;\;B}\rho vdV,\;L=\int_{\;\;B}x\times \rho vdV,\;F=\int_{\;\;B}f_{\text{ext}}dV,\;\text{and}\;\tau =\int_{\;\;B}x\times f_{\text{ext}}dV.$$

Here, B denotes the body region and $f_{\text{ext}}(x,t)$ denotes the external force on the body. The velocity in (and on the surface of) B is given by


(3.15) $$v=v_{0}+\omega \times (x-x_{0}).$$

We define a body-fixed coordinate system such that the representation of a point in the laboratory frame, x , and in the body-fixed coordinates, $\tilde{x}$ , are related by


(3.16) $$\tilde{x}=Q^{T}(x-x_{0})\;\Leftrightarrow \;x=x_{0}+Q\tilde{x}.$$

Here, we have exploited that $Q^{-1}=Q^{T}$ for the orthogonal matrix, Q . Since the body is rigid, the mass density, ρ , in B is constant in time as viewed from the body-fixed coordinates, that is, $\rho =\rho (\tilde{x})$ . The body volume, mass, centre of mass and moment of inertia are, respectively,


(3.17) $$V_{b}=\int_{\;\;B}dV,\;m_{b}=\int_{\;\;B}\rho dV,\;x_{\text{cm}}(t)=\frac{1}{m_{b}}\int_{\;\;B}\rho xdV,\;{\tilde{I}}_{0}=\int_{\;\;B}\rho (\tilde{x})(|\tilde{x}{|}^{2}I_{3}-\tilde{x}\tilde{x})dV.$$

Here, ${\tilde{I}}_{0}$ is the moment of inertia with respect to $x_{0}$ , represented in the body-fixed coordinates.

The equations for linear and angular acceleration are obtained by inserting equations (3.14) [Disp-formula uFD3_14] and (3.17) in (3.13). The resulting equations can be written as


(3.18) $$\left[\begin{array}{cc} m_{b}I_{3} & -m_{b}d_{\text{cm}}\times \\ m_{b}d_{\text{cm}}\times & I_{0} \end{array}\right]\left[\begin{array}{c} {\dot{v}}_{0}\\ \dot{\omega } \end{array}\right]=\left[\begin{array}{c} F+\omega \times m_{b}d_{\text{cm}}\times \omega \\ \tau_{0}-\omega \times I_{0}\omega \end{array}\right],$$

where $d_{\text{cm}}=x_{\text{cm}}-x_{0}$ , $I_{0}=Q\,{\tilde{I}}_{0}\,Q^{T}$ , and $\tau_{0}$ is the torque on the body with respect to the point $x_{0}$ . Together, equations (3.9), (3.12) and (3.18) comprise the rigid body equations of motion. For convenience, we will use the more compact notation


(3.19) $$M=\left[\begin{array}{cc} m_{b}I_{3} & -m_{b}d_{\text{cm}}\times \\ m_{b}d_{\text{cm}}\times & I_{0} \end{array}\right],\;v_{b}=\left[\begin{array}{c} v_{0}\\ \omega \end{array}\right],\;f=\left[\begin{array}{c} F+\omega \times m_{b}d_{\text{cm}}\times \omega \\ \tau_{0}-\omega \times I_{0}\omega \end{array}\right],$$

so that equation (3.18) can be written simply as


(3.20) $$M{\dot{v}}_{b}=f.$$

### 3.3. Added mass

The hydrodynamic force and torque on the body are given by


(3.21) $$F_{h}=\int_{\;\;S}(-pI_{3}+\mu \nabla u)\cdot dS,\;\tau_{h,0}=\int_{\;\;S}(x-x_{0})\times (-pI_{3}+\mu \nabla u)\cdot dS,$$

where S=∂B is the body surface and dS is the differential area vector pointing out of the body region. Because the fluid is incompressible and the body is rigid, the absolute value of the pressure is immaterial. Only variations in pressure along the surface are relevant for the dynamics.

The pressure parts of the hydrodynamic force and torque contain components that are proportional to the instantaneous body acceleration and that go into the total force-torque vector, f , in equation (3.20). Just as we did in the introductory 1-DoF example, we can conceptually split f into a part, $-A\,{\dot{v}}_{b}$ , containing all terms that are proportional to ${\dot{v}}_{b}$ , and another part, $f_{\text{other}}$ , containing all other forces and torques


(3.22) $$f=f_{\text{other}}-A{\dot{v}}_{b},$$

where A is the 6-by-6 added mass matrix, and $f_{\text{other}}\equiv f+A\,{\dot{v}}_{b}$ .

For the added mass matrix, we recall its definition from potential flow theory [31]. In the case of a body moving in an unbounded, incompressible and inviscid fluid, the velocity field can be expressed in terms of a velocity potential, u=∇ϕ . The velocity potential, ϕ , can be decomposed into contributions proportional to the six velocity components (linear and angular) of the body:


(3.23) $$\phi =v_{1}\phi_{1}+v_{2}\phi_{2}+v_{3}\phi_{3}+\omega_{1}\phi_{4}+\omega_{2}\phi_{5}+\omega_{3}\phi_{6}.$$

The functions, $\phi_{1},\ldots ,\phi_{6}$ , are called the unit potentials because they correspond to unit motion along each of the 6 DoF’s. $\phi_{1}$ is found by solving a Laplace equation, $\nabla^{2}\phi_{1}=0$ , requiring that $\nabla \phi_{1}$ approaches zero at infinity and that ${\hat{n}}_{b}\cdot \nabla \phi_{1}={\hat{n}}_{b}\cdot (1\;0\;0{)}^{T}$ m/s on the body boundary. The other unit potentials are found in a similar manner, setting the corresponding body velocity component to one and all others to zero. The added mass matrix can then be expressed in terms of the unit potentials as


(3.24) $$A_{ij}=-\int_{\;\;S}\rho_{f}\phi_{i}(\nabla \phi_{j})\cdot dS,\;i,j=1,...,6.$$

In other words, the first column of the added mass matrix is the linear and angular momentum (or impulse) of the fluid associated with the body moving with unit velocity along the first axis of the coordinate system in which the matrix is represented. Likewise, the second and third column contain, respectively, the fluid linear and angular momentum associated with unit body motion along the second and third coordinate axes. The fourth, fifth and sixth columns are populated with the fluid linear and angular momenta corresponding to unit angular velocity around the three coordinate axes, respectively.

For a body moving in an unbounded fluid, the added mass matrix relative to body-fixed coordinates is constant and entirely determined by the body shape. There are many practical situations where the boundaries are so far away that the domain can be regarded as unbounded.

Even in the presence of viscosity and vorticity, the velocity field can be Helmholtz decomposed into a purely potential part and a purely vortical part [32], and the added mass force on the body can be shown to be unaltered from the potential flow version [33,34]. In other words, the forces and torques on the body from vortices in the fluid—including vorticity in the boundary layer—do not depend on the instantaneous acceleration of the body and hence do not contribute to its added mass coefficients. The independency of the added mass on wake vorticity and on the magnitude of the body acceleration has been numerically verified in [35].

If the domain is bounded, or there are other objects in the fluid, the unit velocity potentials associated with the unit linear and angular motion of the body will still be well-defined but will now depend on the instantaneous geometry of the domain. Hence, as the body moves and reorients relative to the other fluid domain boundaries, the unit potentials and added mass coefficients will also change. Especially if the body pierces a water surface, the instantaneous shape of this surface and the body position and orientation relative to it will influence the added mass matrix. An extreme example is an object falling from air into water, which will give rise to an increase in added mass by a factor of $\rho_{\text{water}}/\rho_{\text{air}}\approx 830$ as the object penetrates the water surface.

## 4. The FloatStepper algorithm

Performing CFD simulations with fixed boundaries, or boundaries moving in a prescribed way, is a standard task performed every day by thousands of engineers and scientists around the world. It is peculiar that adding just 6 DoF’s to the often millions of DoF’s used to represent the fluid state can cause severe numerical difficulties. Of all the infinitely many body paths we could prescribe, exactly one corresponds to the path the body would follow if it was free to move in response to the net forces and torques exerted on it, including hydrodynamic forces and other external forces. It is the job of our coupling algorithm to predict the body acceleration that leads us down this particular path when we use it in our prescription of the body motion. What makes this job so hard is the added mass force and its proportionality to the instantaneous body acceleration with a proportionality constant that we do not know in advance. The most widely used method for solving the implicit acceleration equation is to employ expensive iterations between fluid and body state solvers. Here, we attempt instead to calculate the acceleration directly and non-iteratively. Inserting the decomposed force from equation (3.22) into the body equations of motion, equation (3.20), and isolating the acceleration, we get


(4.1) $${\dot{v}}_{b}=(M+A{)}^{-1}f_{\text{other}}.$$

This equation is well established in linear radiation-diffraction theory for floating bodies [1], where the added mass can be computed at the bodies’ equilibrium position. For it to be useful in unsteady CFD, and without the assumption of small waves or body motion, we need to devise methods to calculate the unknown and time-dependent vector, $f_{\text{other}}$ , and matrix A .

### 4.1. The zero-acceleration step

Let us think of a snapshot of our body-fluid system with body state $\left(x_{b},v_{b}\right)$ , fluid state (ρ,u,p) and possibly a number of external forces acting on the body. At this point in time, we need to decide where the body should go to the next time step in order for its motion to represent free motion. Suppose we took a time step with the same *v*_*b*_ as in the previous time step. This would be experienced by the fluid as a step with zero acceleration, ${\dot{v}}_{b}=0$ . According to equation (3.22), the force experienced by the body during such a zero-acceleration time step would be


(4.2) $$f=f_{\text{other}}.$$

In other words, taking a zero-acceleration time step with our CFD solver and measuring the resulting hydrodynamic response force and torque reveal the non-added mass part of equation (3.21), which, together with gravity, mooring lines, self-propulsion etc., comprises $f_{\text{other}}$ .

### 4.2. Rewinding system

In our process of developing FloatStepper, we initially attempted to take the zero-acceleration time step without actually moving the mesh, as we otherwise do in real CFD time steps. This was, however, found to lead to wrong estimates of $f_{\text{other}}$ . Instead, we take the zero-acceleration time step using mesh motion and exactly the same CFD solver settings as in the real-time step. This ensures accurate estimation of $f_{\text{other}}$ . It also requires a careful time reversal step where, once $f_{\text{other}}$ is obtained, the fluid, body and mesh are brought back exactly to their state before the zero-acceleration time step.

### 4.3. Added mass estimation

To numerically measure the instantaneous added mass matrix, we exploit its definition as the constant of proportionality between hydrodynamic force and body acceleration. We also exploit that the added mass in a viscous fluid with vorticity is identical to the one obtained in the corresponding potential flow situation. In the added mass calculation the convective and viscous terms can be neglected (e.g. [36]), so the equation to solve is simply


(4.3) $$\frac{\partial \rho u}{\partial t}=-\nabla p.$$

We now discretize the time derivative using the Euler scheme,


(4.4) $$\frac{\partial \rho u}{\partial t}\approx \frac{\rho^{n+1}u^{n+1}-\rho^{n}u^{n}}{\Delta t},$$

where the superscript denotes the time step. In potential flow theory, the added mass associated with motion along the x -axis is obtained by impulsively changing the body velocity from zero to $v_{0}=(1,0,0)$ m/s amounting to a boundary condition, $n_{b}\cdot u=n_{b}\cdot (1,0,0)$ m/s for $u^{n+1}$ . Inserting equation (4.4) in equation (4.3) and taking the divergence, we get


(4.5) $$\nabla \cdot \left(\frac{1}{\rho^{n+1}}\nabla (p_{1}\Delta t)\right)=0,$$

where we have used that $u^{n}=0$ , required $\nabla \cdot u^{n+1}=0$ , and marked the pressure with a subscript 1 to indicate that it is the pressure corresponding to acceleration $a_{1}=1$ m/s /Δ⁢t along the first DoF (here chosen to be the x -axis). We have also collected $\Delta \,t\,p_{1}$ in a bracket in equation (4.5) to emphasize that this impulse approaches a constant as Δ⁢t→0 . Physically, this means that if we impose the body velocity over a very short (long) time step, Δ⁢t , then the pressure amplitude required to set the fluid into corresponding motion must be very high (low) such as to keep Δ⁢t⁢p constant. In the limit Δ⁢t→0 , the density $\rho^{n+1}$ approaches $\rho^{n}$ , and so in our added mass calculation step, we do not update the fluid interface position encoded in ρ , that is, we simply use $\rho^{n}$ instead of $\rho^{n+1}$ in equation (4.5).

Once the pressure, *p*_1_, corresponding to the acceleration, $a_{1}=1$ m/s /Δ⁢t of the body along the x -axis is found, the corresponding force and torque on the body are calculated as


(4.6) $$f_{1}=\int_{\;\;S}p_{1}\left[\begin{array}{c} I_{3}\\ (x-x_{0})\times \end{array}\right]dS,$$

and the first column of the added mass matrix is then given by


(4.7) $$A_{1}=-f_{1}/a_{1}.$$

The second to the sixth columns of the added mass matrix are calculated in the same way, calculating the pressure force corresponding to unit body velocity along the other two axes, and unit angular velocity around the three coordinate axes.

In our current implementation of the added mass matrix calculator, the boundary conditions on all other boundaries than the rigid body are copied from the fields used in the real-time step. Solver settings for the pressure equation are also copied from the real-time step pressure solution. The added mass calculator is implemented as a copy of the PISO step in the interFoam solver except that the convective and viscous terms have been removed from the momentum equation. Thus, the calculation is fully parallelized, using the same domain decomposition as the real-time step. The method allows the user to specify which DoF’s should be active in a simulation, for instance, first, second and sixth for a freely moving and rotating body in the x⁢y -plane. It also allows the user to specify a parameter, MaddUpdateFreq, to only update the added mass matrix every MaddUpdateFreq’th time step. This may save computation time in simulations where the added mass is known to only change slowly. Finally, we mention that the added mass calculator class is derived from an abstract base class, allowing future addition of alternative added mass calculator classes (for instance a panel method-based calculator) with runtime selection of the preferred method specified in the case setup files.

### 4.4. Body state update

Once $f_{\text{other}}$ and A have been calculated, as described above, the body acceleration is calculated directly from equation (4.1). This brings us to the actual integration of the body acceleration and velocity to obtain the new velocity and position. This can be done with standard ODE solvers, and while the choice of integration scheme here can have important consequences for energy conservation etc., it is not a point of focus for our work here. We simply use Euler integration, $x^{n+1}=x^{n}+{\dot{x}}^{n}\,\Delta \,t$ , except for the orientation matrix, Q , which we update based on the Rodrigues rotation formula


(4.8) $$Q^{n+1}\approx \left(I_{3}+\sin(|\omega^{n}|\Delta t){\hat{\omega }}^{n}\times +\left[1-\cos(|\omega^{n}|\Delta t)\right]{\left({\hat{\omega }}^{n}\times \right)}^{2}\right)Q^{n},$$

with ${\hat{\omega }}^{n}=\omega^{n}/|\omega^{n}|$ . This ensures that Q stays orthogonal and is exact in the case of constant ω .

### 4.5. Mesh motion

From the fluid side, the body is represented by a boundary patch on which discretized versions of the boundary conditions in §3.1.1 are applied. Thus, after the body position and velocity have been updated to their newly found values, the mesh must follow along. In our current implementation, we use the deforming (or ‘morphing’) mesh functionality of OpenFOAM with the body boundary patch moving rigidly and the mesh points in a region around the patch deforming to accommodate this motion. In this approach, all mesh points closer to the body patch than a user-defined innerDistance follow the body in its rigid body motion. Mesh points outside a user-specified outerDistance from the body patch are kept stationary. Mesh points between these two distances adapt their position smoothly using Spherical Linear Interpolation (SLERP) based on their distance from the body [37]. This leads to acceptable mesh quality as long as the body displacement and rotation are not too large.

### 4.6. Updating boundary conditions on a rigid body

In the meshed fluid domain, the boundary patch representing the rigid body consists of polygonal faces, each with a face centre, $x_{f}$ , which is updated in each time step due to the body motion. We have implemented a velocity boundary field class called floaterVelocity, which holds a reference to a floating body object from which it reads a body position, $x_{0}$ , velocity, $v_{0}$ , and angular velocity, ω . For each face on the rigid body boundary patch, it then sets the velocity field using equation (3.15) with $x=x_{f}$ . The boundary condition takes a boolean parameter called slip. If this is set to true, the boundary condition only takes the normal component from equation (3.15). The velocity component tangential to the face is directly copied from the tangential component of the velocity vector at the centre of the cell to which the face belongs. This is to lowest order (ignoring the curvature of the surface) a symmetry condition on the tangential velocity component.

In the rigid body pressure boundary condition, equation (3.7), the dependency on body acceleration appears indirectly in the first term on the right-hand side. This can be seen by writing it as


(4.9) $$\frac{\partial \rho u}{\partial t}=\rho \dot{v}+v\frac{\partial \rho }{\partial t}=\rho \left[{\dot{v}}_{0}+\dot{\omega }\times (x-x_{0})+\omega \times \{\omega \times (x-x_{0})\}\right]+\left[v_{0}+\omega \times (x-x_{0})\right]\frac{\partial \rho }{\partial t},$$

where we have inserted and differentiated the rigid body velocity, v , from equation (3.15). This acceleration dependency is the very origin of the added mass force on the body and, therefore, is important to capture. We remark that in OpenFOAM, this dependency is treated indirectly by the PISO solution procedure. Discretizing the momentum equation, equation (3.3), it can be written


(4.10) $$a^{u}u^{n+1}=H-\nabla p,$$

where $a^{u}u^{n+1}$ is a collection of all terms proportional to the new time velocity, $u^{n+1}$ , and H (a vector not to be confused with the indicator field introduced in §3.1) contains all other terms except the pressure gradient, ∇⁡p . If the time derivative in equation (3.3) is for instance discretized using the Euler method (equation (4.4)), then $a^{u}$ will contain a term, $\rho^{n+1}/\Delta \,t$ , and H will contain a term, $\rho^{n}u^{n}/\Delta t$ . For boundary faces on the rigid body, these old and new velocities are determined by the specified body acceleration, which is then conveyed to the pressure by imposing a boundary condition for p on the body surface of the form


(4.11) $$n_{b}\cdot \nabla p=n_{b}\cdot \left(H/a^{u}-u^{n+1}\right)a^{u}.$$

Here, for the acceleration to be included correctly, care must be taken when building the $H/a^{u}$ field, and imposing boundary conditions on it. In particular, when assembling the pressure Poisson equation, the standard OpenFOAM solver calls a function, constrainHbyA, which sets $H/a^{u}$ equal to $u^{n+1}$ on boundaries where the u boundary condition is of Dirichlet type, including rigid bodies. This results in $n_{b}\cdot \nabla p=0$ , which we have just argued is incorrect when the body is accelerating. In our simulations, we have found this to give rise to an erroneous behaviour, where the velocity in the cell layer closest to the accelerating body has only around half of the correct magnitude regardless of the thickness of this layer. To avoid this, we make use of the built-in pressure boundary condition called fixedFluxExtrapolatedPressure. Using this, $H/a^{u}$ retains its boundary value obtained as a sum of all the terms from which it is composed, including the previous time velocity, $u^{n}$ , and we no longer observe the erroneous velocity behaviour.

### 4.7. Fluid state update

The exact procedure for solving the fluid equations is not the focus of the work presented here, and will therefore only be described briefly. The implementation is based on OpenFOAM’s interfacial flow solver, interIsoFoam (version v2206) employing the FVM to solve the motion of two immiscible fluids on arbitrary unstructured meshes with cell-centred collocated field representation.

The fluid solver for updating the fluid state, (ρ,u,p) , starts by updating the fluid interface position using the VoF method, where the interface is represented by a volume fraction field expressing for each cell how much of its volume is occupied by the reference fluid. There exist many VoF methods, and the FloatStepper algorithm does not depend on this choice. Here we use the geometric VoF method called isoAdvector, which ensures a sharp interface and accurate, efficient interface advection on arbitrary unstructured meshes [9,38]. The solver can be run in single-phase mode simply by setting the volume fraction field to 1 in all cells and on all boundaries. In this case, isoAdvector will find no interface cells and hence will do nothing.

After the interface advection step the pressure and velocity fields are updated using the PISO algorithm [39]. Details of the OpenFOAM-specific solution procedure can be found in [8,40,41]. When the mesh is moving, the convection of mass and momentum in equations (3.1)–(3.3) is made relative to the mesh motion. This is done by subtracting the flux due to the motion of mesh faces from the physical fluxes across faces in the discretized convective terms, as described, for example, in [42].

### 4.8. Summary of algorithm

In Algorithm 3, we summarize the FloatStepper coupling algorithm. We remark that a similar approach to separating the force into an added mass contribution and everything else was briefly and elegantly sketched by Söding [17]. Our method differs from Söding’s by being non-iterative. The added mass estimate in Söding’s algorithm is found via a minimization process and used as a good initial guess for an iterative solution procedure for the implicit acceleration equation. We, on the other hand, attempt to calculate $f_{\text{other}}$ and A directly and sufficiently accurately, so that iterations can be avoided. Söding provides no validation and only few implementation details. Devolder *et al*. [22] presented a 1-DoF OpenFOAM implementation of a similar iterative approach and showed its favourable stability properties for a heaving floater.


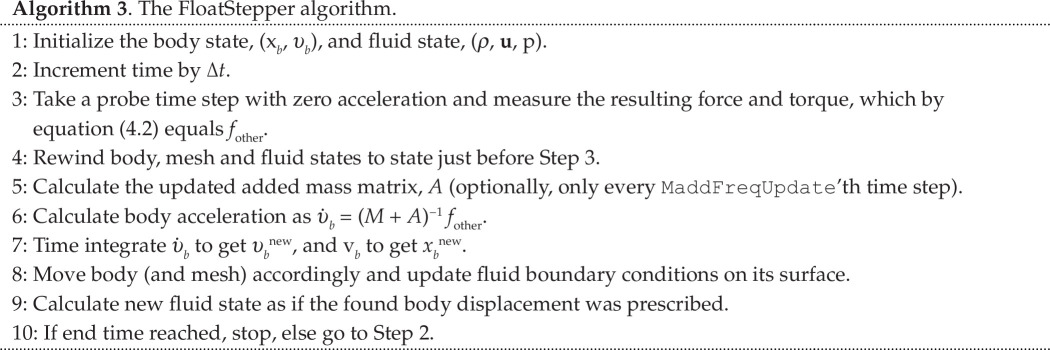


## 5. Validation

### 5.1. Lightweight disc in gravity

In §1, we illustrated the added mass instability with a lightweight disc rising in a heavy, inviscid fluid. We recall that in such an ideal fluid the force on the body is obtained by integrating the pressure over the body surface with the pressure given by the unsteady Bernoulli equation


(4.12) $$p=-\rho_{f}\frac{\partial \phi }{\partial t}-\frac{1}{2}\rho_{f}|u{|}^{2}+C,$$

where C is an arbitrary constant and ϕ is the velocity potential. For pure translational motion along the y -axis, ϕ can be written as $v_{y}\,\phi_{y}$ , where $\phi_{y}$ is the unit velocity potential associated with unit body velocity along the y -axis. The unit potential can be obtained by employing the Milne–Thomson circle theorem [43]. Thus, it is the time derivative of ϕ in equation (4.12), which gives the added mass force contribution proportional to the instantaneous acceleration, ${\dot{v}}_{y}$ , and to $\rho_{f}$ , when integrated over the body surface. Isolating the acceleration in the resulting force expression, one obtains the theoretical, constant body acceleration [31]


(4.13) $$a_{y}=\frac{\rho_{f}-\rho_{b}}{\rho_{f}+\rho_{b}}g_{y}.$$

We remark that the $\rho_{f}$ in the denominator comes from the added mass term and prevents the acceleration from diverging when $\rho_{b}/\rho_{f}\to 0$ . This term is sometimes omitted in the literature, for instance in section 13.10 of *Computational Methods for Fluid Dynamics* by Ferziger *et al*. [44] although their example has $\rho_{b}/\rho_{f}=0.01$ . It is exactly this omission in numerical coupling methods that leads to large overestimation of the body acceleration and numerical instability caused by unphysical kinetic energy injection.

**Figure 4.**
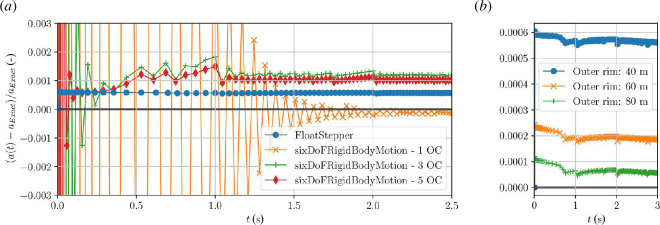
Simulation of the rising disc as described in figure 3. (*a*) Acceleration deviation from theory (equation (4.13)) with sixDoFRigidBodyMotion using 1, 3 and 5 outer correctors (former identical to stable solution in figure 3a)), and with FloatStepper. (*b*) FloatStepper simulation repeated with outer domain boundary at 40 m (same as in (*a*)), 60 m and 80 m.

Figure 4*a* shows the relative acceleration error (relative to *a*_*y*_ from equation (4.13)) as a function of time for a FloatStepper simulation (blue), where the lightweight circle is released to rise buoyantly at time zero. The FloatStepper acceleration is very close to constant with a relative error of around 0.06%. For comparison, we also show in figure 4*a* the results obtained with the sixDoFRigidBodyMotion library of OpenFOAM with 1, 3 and 5 outer correctors in algorithm 2. For those simulations, the initial acceleration is miscalculated due to the initial estimate $a=F/m_{b}$ built into the algorithm. Increasing the number of outer iterations makes the simulation converge faster, but we cannot completely avoid the faulty initial accelerations, and the computational cost increases in proportion to the number of outer correctors.

The sixDoFRigidBodyMotion results in figure 4*a* are run with acceleration relaxation γ=0.8 based on our knowledge of $\gamma_{c}$ from equation (2.7) and figure 3*b* to ensure convergence. We remind the reader that this was only possible because of the simplicity of the case, a circle with known, constant added mass. In practical simulations, the added mass is normally not known and may vary with time. Indeed, one of the frustrating aspects of working with the sixDoFRigidBodyMotion is the guesswork going into setting the acceleration relaxation and the number of outer correctors for a given simulation situation. The user often faces a choice between excessive simulation time and reduced accuracy at best or numerical instability at worst. Eliminating this guesswork is one of the main motivations for developing FloatStepper.

We have numerically investigated the cause of the constant 0.06% error of FloatStepper in figure 4*a*. We have found that the error is unaltered by reducing the time step size or increasing the mesh resolution. The simulation was done with a circle of radius 1 m and with the circular outer rim of the domain placed 40 radii away. We have found that if we repeat the numerical experiment with a domain size of 60 m and 80 m instead of 40 m, the observed deviation from the theoretical value is reduced, see figure 4*b*. This suggests that the deviation from the theoretical (infinite domain) value is, in fact, not a numerical error but rather a finite domain size effect.

### 5.2. Disc in gravity hitting water surface

**Figure 5.**
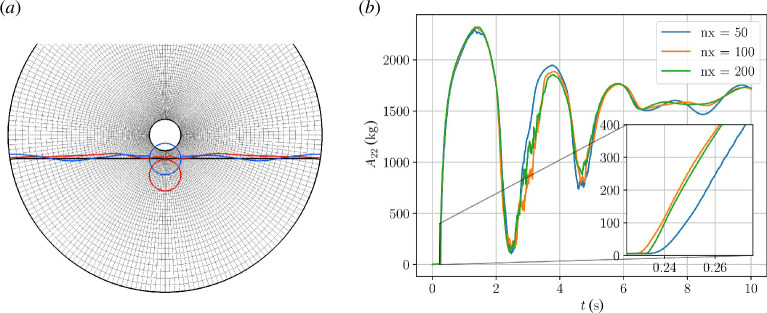
(*a*) A circular domain of radius 10 m centred at the origin and a water surface placed at y=-1.5⁢m . A circular body of radius R=1 m is initialized at the origin with downward velocity $v_{y}=-1$ m/s. Gravity is $g_{y}=-9.81$ m/s^2^, air density is $\rho_{a}=1$ kg/m^3^, water density is $\rho_{w}=1000$ kg/m^3^ and the body has density $\rho_{b}=500$ kg/m^3^. Body position and water surface shown for time t=0 s (black), t=1.3 s (red) and t=10 s (blue). (*b*) Evolution of the vertical added mass component with time for three different mesh resolutions.

In the previous case, we recalculated the added mass at every time step although it was essentially constant due to the long distance to boundaries and the absence of a fluid interface. We now test our added mass calculation procedure with a test case involving a large sudden change in added mass, namely, a circular body falling from air into water. Only the vertical component of the body motion is active. Figure 5*a* shows the initial configuration (black) as well as the body position and water surface at time t=1.3s (red), where the body is fully immersed in water, and at the end of the simulation, t=10s (blue). In figure 5*b*, we show the time evolution of the added mass during the simulation for three different mesh resolutions. As expected, we observe a sudden rise in added mass as the body hits the water surface with a maximum when the body is fully immersed in water. The entry of the body into the water creates waves that are reflected at the domain walls, causing an irregular heaving motion of the body as the added mass settles to its equilibrium value dictated by the density ratios. The convergence with mesh resolution in the initial phase, where the body hits the water surface, is very good as shown in the inset of figure 5*b*. At later stages, the correspondence between the three simulations is also good, although with small variations, presumably due to the difference at different mesh resolutions in the ability to capture the details of the complicated, reflected wave field.

The added mass curves for the fine and intermediate simulations exhibit noisy behaviour near the two local minima around *t* = 2.5s and *t* = 4.7 s. These minima correspond to the disc jumping back out of the water up into the air. Visual inspection of the simulations reveals that a film of water sticks to the disc as it jumps out of the water. The film forms droplets, and as the disc falls back into the water, bubbles are captured beneath it. These interface details are responsible for the erratic features of the added mass curves. The coarse simulation has too large computational cells to capture these interface details, and the added mass curve is, therefore, smoother.

This example demonstrates how the FloatStepper algorithm is able to robustly handle large and abrupt changes in added mass.

### 5.3. Free ellipse in an infinite ideal fluid

When a rigid body free to translate and rotate is immersed in a fluid, the hydrodynamic forces introduce a coupling so that translation can induce rotation and vice verse. It is important to verify that our algorithm captures this coupling correctly. To this end, we consider a benchmark case with a rigid body moving through inviscid fluid with all boundaries far away. According to Howe [33], the hydrodynamic force associated with the body motion can be written


(5.1) $$F_{h}=-\frac{\partial \;}{\partial t}(Tv_{0}+S\omega ),$$

(5.2) $$\tau_{h,0}=-\frac{\partial \;}{\partial t}(S^{T}v_{0}+J\omega )-v_{0}\times (Tv_{0}+S\omega ),$$

where T,S and J are the 3×3 added mass submatrices,


(5.3) $$A=\left[\begin{array}{cc} T & S\\ S^{T} & J \end{array}\right].$$

To see how these added mass coefficients change in time, we note that the matrices T,S and J represented in the body-fixed basis, $\left\{q_{1},q_{2},q_{3}\right\}$ , are constants in time determined solely by the body geometry. We will call these matrices $\hat{T},\hat{S}$ and $\hat{J}$ and note that we have $T=Q\,\hat{T}\,Q^{T}$ and so on. Then, for instance, the first term in equations (5.1) and (5.2) becomes


(5.4) $$\partial_{t}(Tv_{0})=\dot{Q}\hat{T}Q^{T}v_{0}+Q\hat{T}{\dot{Q}}^{T}v_{0}+Q\hat{T}Q^{T}{\dot{v}}_{0}.$$

Noting that $\dot{Q}=\omega \times Q$ and that $(\omega \times {)}^{T}=-\omega \times$ , we can rewrite this to


(5.5) $$\partial_{t}(Tv_{0})=\omega \times Tv_{0}-T\omega \times v_{0}+T{\dot{v}}_{0}.$$

Using this for all the matrix-vector products in equations (5.1) and (5.2), we get


(5.6) $$\left[\begin{array}{c} F_{h}\\ \tau_{h,0} \end{array}\right]=\left(A\left[\begin{array}{cc} \omega \times & 0_{3}\\ 0_{3} & \omega \times \end{array}\right]-\left[\begin{array}{cc} \omega \times & 0_{3}\\ 0_{3} & \omega \times \end{array}\right]A\right)\left[\begin{array}{c} v_{0}\\ \omega \end{array}\right]-A\left[\begin{array}{c} {\dot{v}}_{0}\\ \dot{\omega } \end{array}\right]-\left[\begin{array}{c} 0\\ v_{0}\times (Tv_{0}+J\omega ) \end{array}\right].$$

Inserting this as the force and torque in the body equations of motion, equation (3.18), one obtains the Kirchhoff equations [36,45] for a rigid body moving through an infinite, ideal fluid. They are valuable for evaluating fluid-structure coupling algorithms, like FloatStepper, because they are a set of ODEs that can be solved easily and fast on a computer and encompass non-trivial body-fluid interaction. In fact, for three-dimensional motion, they exhibit chaotic motion [46].

If we restrict ourselves to motion in the infinite x⁢y -plane and choose coordinate axes such that S=0 (always possible because of symmetry of added mass matrix), then equation (5.6) simplifies to


(5.7) $$F_{h}=T(\omega \times v_{0})-\omega \times Tv_{0}-T{\dot{v}}_{0},$$

(5.8) $$\tau_{h,0}=-J\dot{\omega }-v_{0}\times Tv_{0},$$

where now $F_{h}=(F_{x},F_{y},0{)}^{T}$ , $v_{0}=(v_{x},v_{y},0{)}^{T}$ , $\omega =(0,0,\omega {)}^{T}$ and $\tau_{h,0}=(0,0,\tau {)}^{T}$ . For such planar motion, the equations are integrable but still exhibit interesting dynamics and coupling between the 3 DoF’s. In particular, from equation (5.7), we see how the hydrodynamic force depends not only on instantaneous body acceleration but also on its velocity when ω≠0 . Similarly, equation (5.8) shows how the torque with respect to $x_{0}$ depends on the translational velocity, $v_{0}$ (except for steady-state motion along the principal axes of T ).

Many useful coupling validation cases can be constructed based on the Kirchhoff equations and their solutions. Here, we consider a body of elliptic shape with major and minor axes $R\,\left(1+b^{2}\right)$ and $R\,\left(1-b^{2}\right)$ , respectively, where the shape parameter b∈[0,1] . For such a body, the added mass coefficients for motion along major axis, minor axis and for rotation are, respectively,


(5.9) $$A_{11}=\rho_{f}\pi R^{2}(1-b^{2}{)}^{2},\;A_{22}=\rho_{f}\pi R^{2}(1+b^{2}{)}^{2},\;A_{66}=2\rho_{f}\pi R^{4}b^{4}.$$

**Figure 6.**
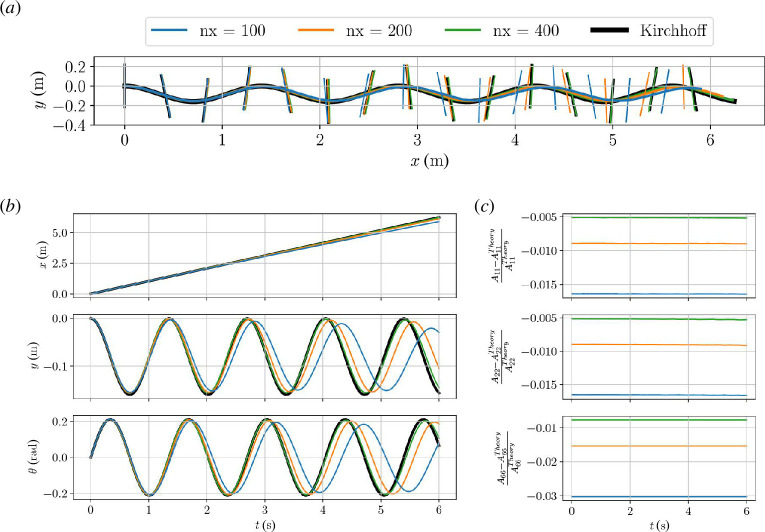
(*a*) Trajectory of an elliptic body of density $\rho_{b}=0$ kg/m^3^ moving through an infinite two-dimensional ideal fluid of density $\rho_{f}=1$ kg/m^3^. Initial body centre at (0,0) with body minor axis aligned with the x -axis. Initial velocity is $\left(v_{x},v_{y},\omega \right)=\left(1,0,1\right)$ . Black curve is exact solution while simulations with three different mesh resolutions are shown in colours. Body orientation (major axis) shown at 15 locations along the trajectory. (*b*) Body coordinates as function of time. (*c*) Relative deviation of added mass coefficients from infinite domain values in equation (5.9).

In our numerical experiment, we use R=1 m, b=0.5 , fluid density $\rho_{f}=1$ kg/m^3^ and body density $\rho_{b}=0$ kg/m^3^. The body is initialized with its centre at the origin and its minor axis aligned with the x -axis. The initial velocity is chosen to be $v_{x}=1$ m/s, $v_{y}=0$ m/s and ω=1 rad/s, which gives rise to an undulatory motion along the x -axis while the body wiggles with an angular amplitude of around 11°. Our simulation is started at t=0 s and ends at time t=6 s corresponding to around 4.5 motion periods. Adaptive time stepping was used with a maximum Courant–Friedrichs–Lewy (CFL) number of 0.1. The circular outer rim is placed at 40⁢R . Simulations were done with three different mesh resolutions with 100 , 200 and 400 cells in the radial direction with grading such that the inner most cells are 50 times smaller than the outer most cells. The corresponding azimuthal resolutions were 120 , 240 and 480 cells. Simulations obtained with the three mesh resolutions are shown in figure 6*a* together with the exact solution obtained by integrating the Kirchhoff equations directly. Figure 6*b* shows the horizontal, vertical and angular body coordinates and their convergence to the exact solution (black curves) with mesh refinement. The added mass coefficients are recalculated in each time step. Figure 6*c* shows the relative error in the added mass coefficients as a function of time with respect to equation (5.9). The added mass is very close to constant throughout each simulation, and the error is seen to diminish with increased mesh resolution. As in the rising circle case, it is possible that some of this error is due to the finite domain size.

### 5.4. Freely floating box in regular waves

**Figure 7.**
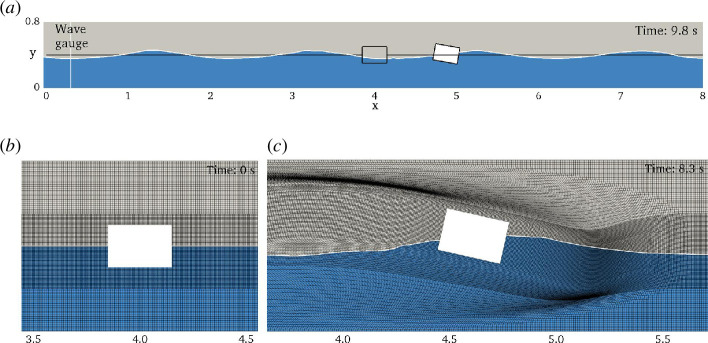
(*a*) Snapshot of free-floating box case from Ren *et al*. [47] with H=0.10 m and T=1.2 s at time t=9.8 s simulated with the coarse mesh and CFL ≤ 0.5. Water volume is shown in blue and water surface and box in white. The initial surface and box position are shown for reference. (*b*) Fine mesh near box at t=0 s. (*c*) Fine mesh deformation at time t=8.3 s.

We now increase the level of complexity by considering a case combining a free surface with several active DoF’s. We choose the benchmark case presented in Ren *et al*. [47] with a box floating freely in regular waves in a wave flume. Here, we try to reproduce their experimental data, including recorded surface elevation and box surge ( x ), heave ( y ) and pitch ( θ ) motion. The physical wave flume is 23 m long, 44 cm wide and filled to a water depth of d=40 cm. The floating box is 30 cm long, 20 cm high and 40 cm wide, leaving a gap of 2 cm to each side wall of the flume. The box is made of 8 mm thick Perspex plates and has a compartment in the middle filled with a granular material to give it an overall density of 500 kg/m^3^, while retaining its centre of mass at its geometric centre. The total mass of the floater is then 12 kg. Assuming the density of Perspex to be 1180 kg/m^3^ and that the granular filling material is evenly distributed in the inner cross-sectional area of the box, we calculate the moment of inertia with respect to its long centre axis to be $I_{\text{box}}=0.151$ kg m^2^. The box is initialized in equilibrium, half immersed in water ( $\rho_{w}=1000$ kg/m^3^), with its centre 2 m from a piston-type wave-generating wall placed at one end of the flume. To minimize wave reflections, a wave absorber is placed at the opposite end of the flume. Ren *et al*. [47] perform two free floater tests with regular waves of wave height H=0.04 m and H=0.10 m, respectively, both with wave period T=1.2 s. No detailed information is provided about the type of waves produced. In our numerical setup, we generate waves using a custom-made piston-type wave maker. It works by squeezing and stretching the cells in a region in front of the wave piston wall such as to make the piston wall move in accordance with a user-supplied displacement file. The piston displacement signal we use is given from wave piston theory by


(5.10) $$X(t)=-H\frac{\sinh(kd)\cosh(kd)+kd}{4\sinh^{2}(kd)}\sin(\omega t),$$

where ω=2⁢π/T , and k is found numerically by solving the dispersion relation,


(5.11) $$\omega^{2}=gk\tanh(kd),$$

for the given choice of wave period, T , water depth, d , and gravity, g . Given the two-dimensional nature of the problem with only a small gap between the box and flume walls, we choose to model the experiment in a two-dimensional setup. We choose a domain height of 0.8 m with the initial horizontal water surface in the middle at y=0 m. To limit the mesh size, we truncate the flume 8 m from the wave-piston wall and use the active wave absorption boundary condition built into OpenFOAM to minimize wave reflections [48,49]. The cells in our coarse base mesh are squares with a side length 1 cm, giving a mesh size of 63 400 cells. We also use a fine mesh with the region between y=0.2 m and y=0.55 m (covering the water surface and the box) refined into squares of side length 0.5 cm giving a total of 145 600 cells. Figure 7*a* shows the initial non-deformed fine mesh near the box. The simulations are run on the 16 cores of an AMD EPYC 7301 processor with time steps adjusted to keep the maximum CFL number in the domain below 0.5. On the coarse mesh numerical experiments were also done with CFL < 0.1. Snapshots of box position and water surface at time t=0 s and t=9.8 s are shown in figure 7*a* for the H=0.10 m case.

**Figure 8.**
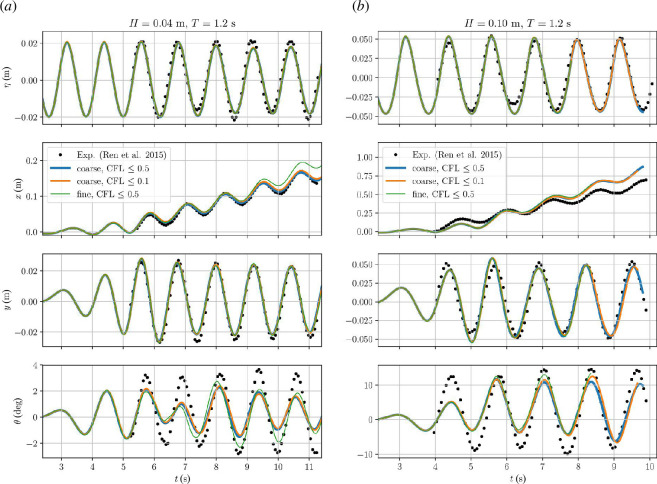
Freely floating box experiments from Ren *et al*. [47] with regular waves of height (*a*) H=0.04 m and (*b*) 0.10 m. Surface elevation measured 0.3 m from wave piston (first row), body surge (second row), heave (third row) and pitch (fourth row) motion shown for FloatStepper simulations with three different resolutions.

Ren *et al*. [47] provided experimental data for five wave periods on the interval t∈[0, 6] s. This data is plotted with black dots in figure 8 and shows almost periodic motion with ∼ 15% variation in amplitude between largest and smallest wave. No data or information are given about the preceding time interval where the waves and body motion were building up. Unless the box was kept fixed during this ramp-up period, it will have drifted some distance from its initial position at x=2 m. There is, therefore, some uncertainty about the offset for the surge motion shown in the second row of figure 8. In our numerical experiments, we have observed that this distance may be of importance because the waves reflected from the box interact with the incoming waves in the region between the wave piston and box. Thus, if we start our box at x=2 m, we generally overestimate the surge drift. Domínguez *et al*. [50] have also tried to numerically reproduce the results in [47] and have successfully reproduced the surge drift with only slight overestimation in their highest resolution simulation. They do not explicitly mention their starting position for the box but from their figures, we infer that they started it at x=4 m. We will, therefore, use this starting position in our simulations.

Our FloatStepper results for the two mesh and time resolutions are shown in figure 8. For the free surface elevation in the top row, Ren *et al*. [47] do not mention where in the domain it is recorded. We have chosen to record the surface elevation 0.3 m in front of the wave maker. We compensate for the phase difference caused by the different numerical and experimental wave gauge positions by shifting the experimental wave data by 4.4⁢T along the time axis for the H=0.04 m case, and by 3.4⁢T for the H=0.10 m case. The results demonstrate a good match in wave height and period between the experimental data and all three numerical runs.

The second row of panels in figure 8 shows the box surge motion that is characterized by oscillations superimposed on a steady drift in the direction of wave propagation. For the H=0.04 case, the coarse simulations with CFL = 0.1 and 0.5 capture both oscillations and drift very well. The fine simulation overestimates the drift for the last two periods. We do not currently have a good explanation for this behaviour. For the H=0.10 m case, all three simulations give virtually identical surges but with an overestimation of the drift motion in all five wave periods. As mentioned, this may be due to differences in horizontal initial box position. We note that the accumulated drift of around 0.7 m during the five wave periods causes significant mesh distortion with the currently available SLERP-based mesh deformation in OpenFOAM. The two coarse meshed simulations crash at around t=9.7 s due to this, and the fine-meshed simulation crashes after t=8.3 s. Figure 7*c* shows the mesh deformation just before the crash. In future work, we will incorporate an improved mesh deformation method that allows for larger lateral displacement without compromising mesh quality. We also plan to couple FloatStepper with the overset mesh implementation in OpenFOAM, which will allow for arbitrarily large body displacements and rotations without the problem of deteriorating mesh quality.

The heave motion of the box is shown in the third row of figure 8. This is captured very well for both wave heights with virtually no difference between the simulations with different mesh and time resolution.

We show the pitch motion of the body in the last row of figure 8. For both wave heights, the experimental pitch data is characterized by oscillations with 15–20% variation in amplitude. All our simulations underestimate the amplitude of the pitch oscillations. For the H=0.04 case, the numerical oscillations have irregular amplitude with minor but noticeable differences between the coarse and fine simulations. For the H=0.10 m case, the simulated pitch oscillations are more regular but with a slightly longer period than the experiments. This may be due to the overestimated numerical surge drift, causing a Doppler-like shift in the period of the pitch forcing from the incoming waves.

The missing experimental details about wave generation and ramp-up make it difficult to draw firm conclusions about the origin of our underestimated pitch. Both [47] and [50] obtain a better pitch match in their SPH simulations, although with a slight tendency to overestimate the amplitude. A possible explanation for our deviations in pitch amplitude could be the cell skewness developing due to the surge drift (figure 7*c*), an issue that does not exist for the meshless SPH method. This will be further investigated with the new mesh deformation method to be developed.

### 5.5. Moored floating box in regular waves

**Figure 9.**
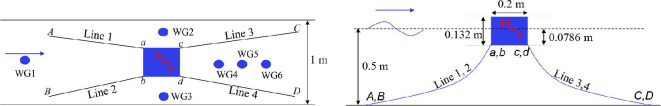
Numerical setup for the moored floating box experiment [51], including the fairlead and anchor points denoted as [a, b, c, d] and [A, B, C, D], respectively. Bottom figure represents the wave gauge positions (WG) around the box (not to scale). The coordinates for these points are provided in table 1.

In our last benchmark case, we validate FloatStepper against experimental data for a moored floating box in regular waves using data from the MaRINET2 EsflOWC project [51]. The physical tests were conducted in a 30 m long and 1 m wide wave flume with a water depth of 0.5 m. Box dimensions, mooring configuration and wave gauge positions are shown in figure 9 and listed in table 1. To track the surge, heave and pitch motion of the box, a wooden plate with light-reflecting markers was attached to the front of the box. The tension in the four mooring chains was also recorded during experiments.

**Table 1.** Box and mooring parameters along with coordinates of the mooring line anchor and fairlead connections from the experiment [51].

box properties		mooring lines		wave gauges (x,y) [m]	
box length	0.2 m	mooring diameter	0.003656 m	WG1	(−2.74, 0.00)
box width	0.2 m	mooring weight	0.0607 kg/m	WG2	(−0.05, 0.26)
box height	0.132 m	mooring length	1.455 m	WG3	(0.07, −0.36)
box weight	3.148 kg	axial stiffness	29 N	WG4	(0.55, 0.00)
centre of gravity	(0, 0, −0.0126)	fairlead a,b,c,d	(±0.1,±0.1, −0.0736)	WG5	(1.90, 0.00)
box draft	0.0786 m	anchor A,B,C,D	(±1.385, ±0.423, −0.5)	WG6	(2.90, 0.00)

In our numerical setup, we use a shortened three-dimensional flume domain of length 10 m covered by a mesh of approximately 5 million cells. Simulations were run on 100 cores of an AMD EPYC based HPC cluster.

To model the mooring lines, we have coupled FloatStepper with MoorDyn [52], an open-source library designed to couple dynamic mooring line dynamics with rigid body solvers. MoorDyn includes catenary moorings, seabed friction, axial and bending stiffness, hydrodynamic drag and mooring line-added mass effects. The coupling with FloatStepper follows the methodology presented in [53], where the motion solver passes the floater position and velocity to MoorDyn, which calculates and returns the net mooring restraining forces and moments from all fairlead tensions.

The experimental data from MaRINET2 EsflOWC reported in the literature [50,54,55] contains three different combinations of wave height and period. We have run all three cases and found a similar degree of correspondence between experiments and FloatStepper results in all of them. Here, we therefore only show the case with a wave height of 0.12 m and a wave period of 2 s (Case 3 from [50] and Case 2 from [54]). The case was run for 20 simulation seconds corresponding to 10 wave periods with a maximum CFL number limit of 0.5. Different turbulence models were tested in [54] for the same test case, and it was found that the choice of turbulence model had minimal impact on the results, and hence, no turbulence model was applied in this simulation.

**Figure 10.**
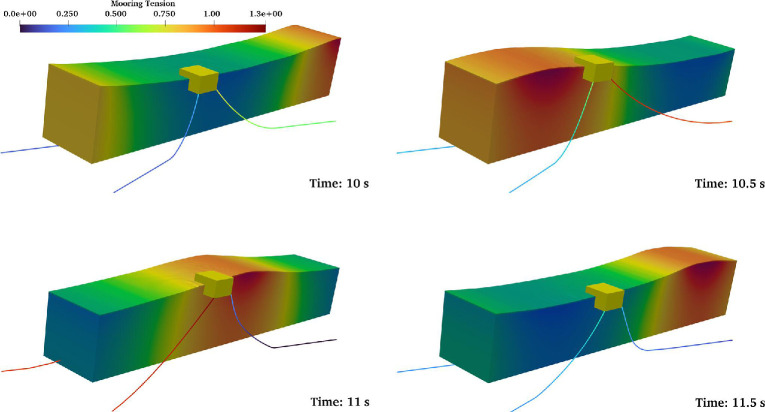
Instantaneous pressure distribution and a catenary mooring system configuration over one wave cycle for the box interacting with regular waves ( T=2 s, H=0.12 m).

**Figure 11.**
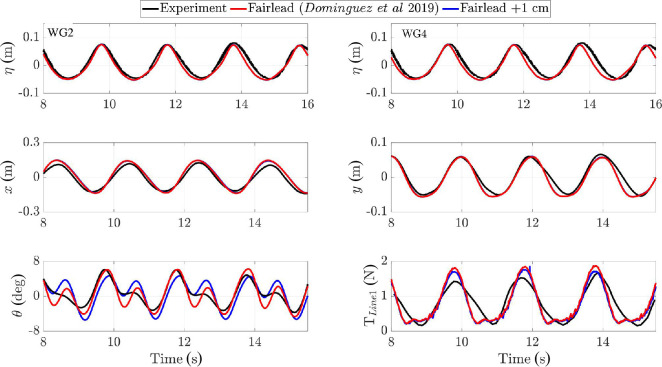
Moored floating box exposed to regular wave with T=2 s and H=0.12 m. Surface elevation ( η ) shown at WG2 and WG4, body surge ( x ), heave ( y ), pitch ( θ ) and tension in mooring line 1 $\left(T_{Line1}\right)$ . Experimental data (black) taken from [50]. FloatStepper simulations performed with reported fairlead positions (red) and with fairleads moved 1 cm up (blue) to illustrate large pitch sensitivity to this position.

Figure 10 depicts snapshots during a single wave period, showing body displacement, dynamic fluid pressure and mooring line shapes and tension. Figure 11 presents a comparison of experiments (black) and CFD (red) for surface elevation at WG2 and WG4, box surge ( x ), heave ( y ), pitch ( θ ) and fairlead tension in Line 1 ( $T_{L\,i\,n\,e\,1}$ ). There is a reasonable agreement in surface elevation, heave and surge motion although we observe a slight overestimation of the latter. In terms of pitch motion, our simulation captures the overall amplitude and phase but overestimates the secondary peak amplitude between the main peaks. Similar pitch deviations were found in earlier numerical studies based on both FVM and SPH [50,54,55], where it was suggested that the deviations could be due to the mounted wooden plate altering the inertial properties of the floater. We have numerically investigated the effect of changing the centre of gravity, the body inertia and the mooring parameters to better understand the observed discrepancies. We have found that the pitch motion is very sensitive to variations in the fairlead point. This is illustrated in figure 11 with the blue curves, where we moved the fairlead attachment point 1 cm up on the box and repeated the simulation. This has virtually no effect on surface elevation, surge and heave (blue curves overlapped by red) but leads to significant differences in pitch motion and in the maximum mooring tensions. We conclude that it is vital to have precise fairlead position data from experiments to be able to reproduce pitch motion for the moored floating box case.

In summary, while the details in pitch motion require more investigation and validation, the overall translational and rotational behaviour was well-captured by the solver.

## 6. Summary and discussion

We have demonstrated the feasibility of a new coupling algorithm, FloatStepper, for FVM-based CFD simulation of an incompressible fluid and a floating rigid body. The method is based on direct calculation of the instantaneous added mass matrix, which allows for the separation of the added mass force from the other hydrodynamic forces. Hereby, the equations of motion can be solved robustly without iteration. While other researchers have previously proposed to introduce explicit added mass calculation, the combination of direct evaluation, non-iterative form and accessibility in a widely used open-source CFD software framework is a novelty of our work.

The robustness of the algorithm has been demonstrated through five simple test cases. First, for a rising disc in unbounded fluid, the solver is able to determine the acceleration with a relative error of less than 0.01%. Next, for a disk falling into flat water, the solver is able to handle the abrupt change in added mass by a factor of $\rho_{w}/\rho_{a}=830$ at the initial entry, and we demonstrated convergence with mesh refinement. The hydrodynamic coupling between translational and rotational DoF’s was tested against a benchmark with a wiggling ellipse travelling through unbounded fluid, where the Kirchhoff equations provide an exact solution. For this test, a zero body mass was used to demonstrate the absence of added mass instability, and the solver was shown to converge to the analytical solution upon mesh refinement.

The solver performance for floating structures in waves was next benchmarked in two test cases with a box floating freely and exposed to regular waves. Body motion was found to be reasonably well predicted with only a small dependency on mesh and time resolution but with some overestimation of surge drift for the case with the largest wave height and underestimation of pitch amplitude for both cases. Some of the observed discrepancy may be ascribed to the lack of an exhaustive description of the experimental setup details such as wave and floater behaviour prior to the recorded experimental time interval.

Our last test case included a coupling with the MoorDyn library to compute the motion of a moored box in regular waves. The surge was well matched while the pitch motion deviated through a larger amplitude of a secondary motion peak in between the main peaks. The mooring line tension amplitude was overestimated by around 15%. For this experiment, some uncertainty for the mass properties of the setup has been described by other researchers, who have discussed similar deviations. For both floating box cases, the pitch comparison with experiments is only partially satisfactory, and there is both a need for further validation of the code and for more exhaustively described experimental data for this kind of validation cases. We are currently running new experiments for an offshore wind floater in waves and will validate FloatStepper against these in forthcoming publications.

The current FloatStepper implementation in OpenFOAM is published as open-source [26] (including setup files for all validation cases presented here) in the hope that it will be used and extended by the CFD community, scientists and engineers working with floating objects. The shared code is at a proof-of-concept level of maturity. This means that, while it can certainly be used for production CFD runs for floating object simulations, there is still room for improvement. In particular, the code can be optimized in terms of both speed and memory usage. A central aspect here is the explicit added mass calculation. This comes at the price of solving six Poisson equations in the full domain but eliminates the need for outer corrections, and—just as importantly—the uncertainty associated with choosing a safe, yet efficient, value for the acceleration relaxation parameter. In principle, FloatStepper with six active DoF’s will cost the same as running with eight outer correctors (one zero-acceleration time step and six added mass column calculations in addition to the real-time step). However, our experience so far is that an added mass column calculation is not nearly as expensive as the full PISO time step of an outer corrector iteration. A quantification of this computational cost difference will be the subject of further studies, where we will also investigate the effect on accuracy and efficiency of reducing the added mass updating frequency.

Several numerical aspects, such as the ODE solver for the 6-DoF update, the interface advection method and the type of mesh morphing, are currently hardcoded in the FloatStepper implementation. We plan to extend the code to allow the user various choices of schemes and methods and to easily add and test own customized methods. An important future extension would be to couple the method with overset mesh and immersed boundary methods to allow more extreme body motions than what is feasible with the deforming mesh method. Another relevant extension area would be the ability to handle multiple rigid bodies, which would enable simulations, for example, of the interaction of an installation vessel with a floating offshore wind turbine foundation.

A robust floating body algorithm is a prerequisite for realizing the full potential of CFD as an engineering tool within fluid–structure interaction. We hope that the open-source release of FloatStepper will help realize this potential and foster collaboration in the CFD community to further improve the predictive capabilities of floating body CFD.

## Data Availability

The FloatStepper OpenFOAM implementation and all case setup files used to generate the data and figures presented in this article are available in the GitHub repository www.github.com/FloatStepper/FloatStepper and archived on www.zenodo.org [26].
